# Association between depression and infertility risk among American women aged 18–45 years: the mediating effect of the NHHR

**DOI:** 10.1186/s12944-024-02164-3

**Published:** 2024-06-10

**Authors:** QiaoRui Yang, Jing Tao, Xin Xin, JinFu Zhang, ZhenLiang Fan

**Affiliations:** 1 Department of Gynecology, Guanghua Hospital Affiliated to Shanghai University of Traditional Chinese Medicine, Shanghai, China; 2 Shanghai University of Traditional Chinese Medicine, Shanghai, China; 3 Heilongjiang University of Chinese Medicine, Heilongjiang, China; 4 Department of Gynecology, Shenzhen Hospital of Shanghai University of Traditional Chinese Medicine, Guangdong, China; 5 Nephrology Department, The First Affiliated Hospital of Zhejiang Chinese Medical University (Zhejiang Provincial Hospital of Chinese Medicine), Zhejiang, China; 6 Academy of Chinese Medical Science, Zhejiang Chinese Medical University, Zhejiang, China

**Keywords:** Infertility, Depression, NHHR, NHANES, Lipid metabolism

## Abstract

**Background/Objective:**

Depression and infertility are major medical and social problems. The non-high-density lipoprotein cholesterol to high-density lipoprotein cholesterol ratio (NHHR) serves as an innovative and reliable lipid marker for cardiovascular disease risk assessment. Previous research has indicated a potential correlation among lipid metabolism, depression, and infertility. Nonetheless, the exact involvement of lipid metabolism in modulating the pathological mechanisms associated with depression-induced infertility remains to be fully elucidated. The aim of this study was to explore the connection between depression and infertility and to assess whether the NHHR mediates this association.

**Methods:**

A cross-sectional analysis was performed utilizing data from there cycles (2013–2018) of the National Health and Nutrition Examination Survey (NHANES) database. Female infertility was assessed according to the responses to the RHQ074 question in the reproductive health questionnaire module. Depression states were evaluated using the Patient Health Questionnaire-9 and classified into three grades based on the total scores: no depression (0–4 points), minimal-to-mild depression (5–9 points) and moderate-to-severe depression (10 or more points). The NHHR was calculated from laboratory cholesterol test results. Baseline population characteristics were compared, and subgroup analyses were carried out based on the stratification of age and body mass index (BMI). Weighted multivariable logistic regression and linear regression models, with adjustments for various covariables, were employed to examine the associations among depression, infertility and the NHHR. Finally, mediation analysis was utilized to explore the NHHR's potential mediating role in depression states and female infertility.

**Results:**

Within this cross-sectional study, 2,668 women aged 18 to 45 years residing in the United States were recruited, 305 (11.43%) of whom experienced infertility. The study revealed a markedly higher prevalence of depression (*P* = 0.040) and elevated NHHR (*P* < 0.001) among infertile women compared to the control cohort. Furthermore, moderate-to-severe depression states independently correlated with increased infertility risk, irrespective of adjustments for various covariables. Subgroup analysis indicated a positive association between depression and infertility risk within certain age categories, although no such relationship was observed within subgroups stratified by BMI. The findings from the weighted logistic regression analysis demonstrated that the elevated NHHR is positively associated with heightened infertility risk. Additionally, the weighted linear regression analysis indicated that moderate-to-severe depression is positively linked to the NHHR levels as well. Finally, the association between depression states and female infertility was partially mediated by the NHHR, with the mediation proportion estimated at 6.57%.

**Conclusion:**

In the United States, depression is strongly correlated with an increased likelihood of infertility among women of childbearing age, with evidence suggesting that this relationship is mediated by the NHHR. Subsequent research efforts should further explore the underlying mechanisms connecting depression and infertility.

**Supplementary Information:**

The online version contains supplementary material available at 10.1186/s12944-024-02164-3.

## Introduction

Infertility is a medical condition characterized by the inability to achieve a successful pregnancy outcome following 12 months or more of regular and unprotected sexual intercourse or due to an impairment in an individual's or partner's reproductive capacity [1, 2]. The worldwide incidence of infertility is estimated to range between 8 and 12%, indicating that approximately one out of every eight couples will receive medical intervention [3, 4]. In recent years, infertility has garnered attention as a noteworthy public health issue on a global scale, exerting profound effects on individuals, families and society as a whole [1]. The implications of infertility extend beyond the realm of involuntary childlessness and encompass various public health ramifications, such as psychological distress, marital discord, social stigmatization and economic burden [5].

Mental disorders have a significant impact on the global burden of disease. According to a report released by the World Health Organization (WHO) in 2019, approximately 970 million people around the world are afflicted by mental disorders, with depression recognized as one of the prevalent manifestations [6]. Additionally, insights from the 2019 Global Burden of Disease Study highlighted that depression ranks among the foremost causes of disability, affecting around 280 million individuals annually and resulting in disability for more than 47 million individuals [7, 8]. Of particular concern is the higher prevalence of depression among women facing infertility compared to those who are fertile [9]. According to a meta-analysis, the total prevalence of depression among infertile women is 44.32% in low- and middle-income nations and 28.03% in high-income countries [10]. Depression and infertility have a bidirectional link. The experience of infertility, coupled with the uncertainty of treatment options, financial burdens and social prejudice, contributes to heightened psychological stress among infertile women, which in turn could act as an underlying risk factor for infertility, ultimately seriously impacting the overall physical and mental health of women [11].

Cholesterol is essential for both male and female fertility. It has been proven that the depletion of cholesterol is correlated with reduced fertilization rates and index, along with delayed extrusion of the second polar body [12]. However, these pathological changes were reversed by cholesterol supplementation [12]. Conversely, high cholesterol levels seem to have a detrimental effect on male fertility [13]. Cholesterol overload causes eggs to escape from metaphase II arrest and activate prematurely, which eventually disrupts the normal synchronization between fertilization and meiotic completion, resulting in infertility [14]. Apart from its crucial function in reproduction, cholesterol also exerts prominent influence within the brain's second messenger system, a mechanism implicated in the biological mode of action of mood stabilizers and antidepressants [15, 16]. The non-high-density lipoprotein cholesterol to high-density lipoprotein cholesterol ratio (NHHR) is an emerging lipid ratio that has been identified as a valuable tool for assessing the atherogenic lipid profile [17]. The NHHR provides insight into the composition of various lipoproteins and has been linked to a number of health status, containing metabolic syndrome, diabetes, depression, cardiovascular and cerebrovascular diseases as well as other disorders [18–21].

Given the multifaceted roles of cholesterol in both reproduction and the brain, it is important to further explore the potential role of the NHHR in depression and infertility. However, a paucity of evidence persists regarding the association between depression states and female infertility risk, particularly concerning the cholesterol levels' impact within this domain. This research marks the first attempt to investigate the mediating effect of the NHHR, a novel proxy measure of cholesterol levels, on the connection between depression states and female infertility, utilizing data sourced from the National Health and Nutrition Examination Survey (NHANES) database, aiming to provide fresh evidence regarding the interplay among depression, the NHHR, and infertility within the US population.

## Methods

### Study design

The NHANES, conducted by the National Center for Health Statistics (NCHS) in the United States, aims to evaluate the status of health and nutrition for both adults and children throughout the entire nation, covering a broad spectrum of domains including demographics, socioeconomic factors, dietary behaviour and health-related inquiries. Comprising two parts—an interview and a physical examination—the NHANES was administered by highly trained medical professionals, conducted either at participants' residences or within a mobile examination centre. The study protocol for the NHANES received approval from the NCHS Ethics Review Board of the Centers for Disease Control and Prevention, ensuring adherence to ethical guidelines. Prior to their involvement in the survey, written informed consent was obtained from each participant.

**Fig. 1:**
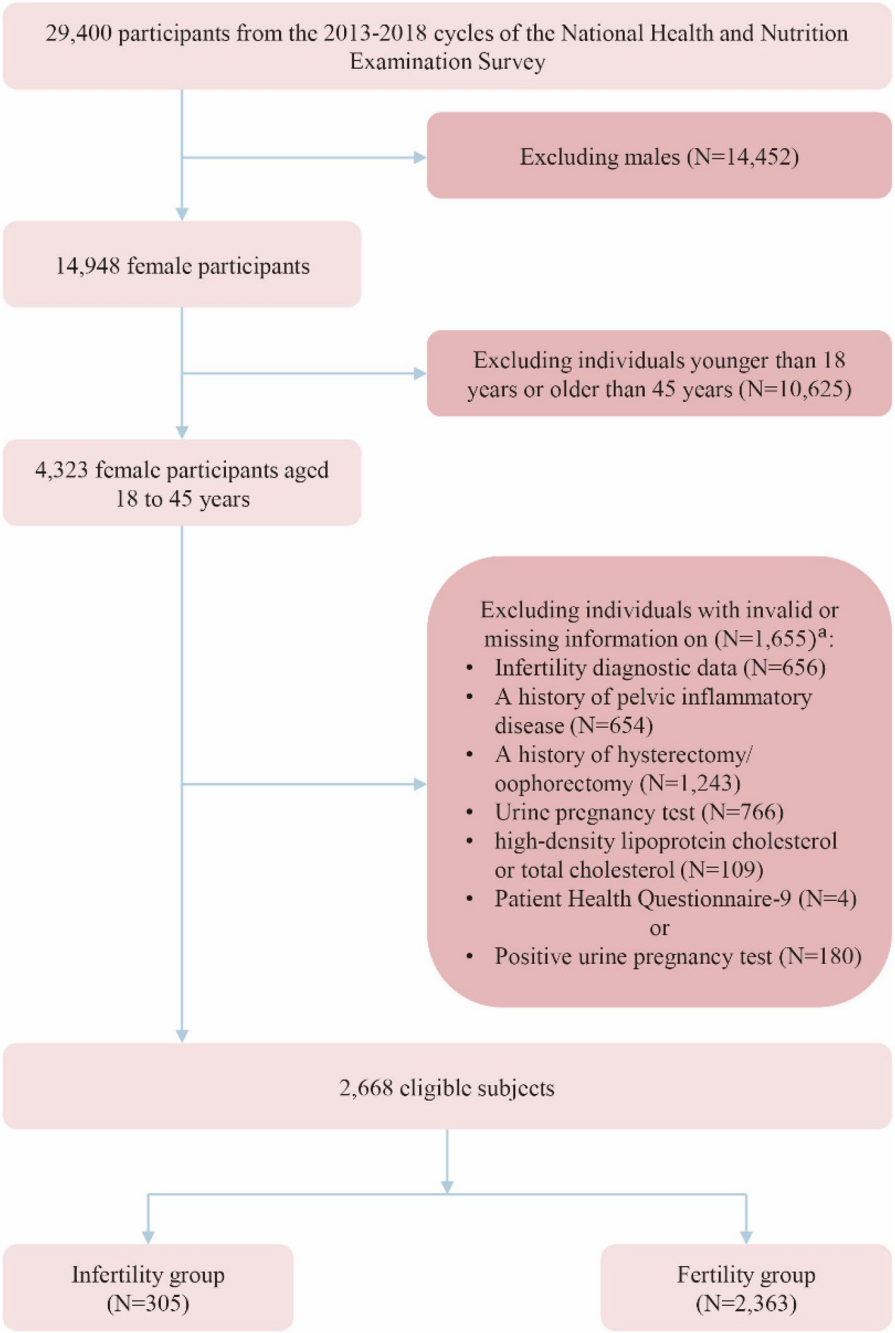
The flowchart of the study populations screened from the 2013–2018 NHANES cycles. ^a^ There is an overlap between the populations

Datasets from the NHANES cycles of 2013–2014, 2015–2016 and 2017–2018 were utilized in this study, as these were the only three cycles for which complete relevant data were available. Initially, 29,400 subjects were enrolled, containing 4,323 females aged 18 to 45 years. Rigorous criteria were applied to exclude subjects from the analysis. This involved excluding individuals who had missing information on the infertility questionnaire, those who refused to answer the questions and those who were unaware of the answers. Moreover, individuals with positive or missing results in the urine pregnancy test were also excluded, as were those with a history of hysterectomy or oophorectomy or an absence of relevant information regarding these procedures. Additionally, those without a history of pelvic inflammatory disease or without complete cholesterol (TC) or high-density lipoprotein (HDL) data were not taken into consideration for inclusion. Finally, individuals with missing information on the depression questionnaire were also excluded. After the screening process, a final sample size of 2,668 eligible subjects remained for the follow-up analysis. Among them, 305 were identified as infertility patients, while 2,363 served as controls. The detailed screening process is visually presented in Fig. 1.

### Assessment of infertility

Self-reported infertility was defined using the reproductive health item in the questionnaire data module (RHQ074). Individuals who responded affirmatively ("Yes") were categorized into the infertility group, while those who responded negatively ("No") were allocated to the control group. Participants who declined to answer or were unaware of their status were systematically excluded from the analysis to ensure the integrity and accuracy of the findings.

### Evaluation of depression states

The Patient Health Questionnaire-9 (PHQ-9) was utilized to evaluate the participants' depression states in the mental health section of the questionnaire data module. Comprising nine items, the PHQ-9 serves as a screening tool intended to assess the degree of depression that a person has experienced during the last two weeks. It also provides valuable insights into a variety of issues related to the emotional well-being of the participants. Participants' responses to each item on the PHQ-9 were recorded on a scale from "not at all" (0 points) to "nearly every day" (3 points), allowing for an assessment of depression levels. The scores from each participant's answers to the nine questions were then summed to calculate their depression score, which ranged from 0 to 27. These scores were subsequently categorized into three levels: no depression (0–4 points), minimal-to-mild depression (5–9 points) and moderate-to-severe depression (10 or more points) [22].

### Calculation of the NHHR

The following equation was used to determine the NHHR based on the fasting participants' lipid profiles: NHHR = non-HDL-C/HDL-C, where non-HDL-C = TC-HDL-C. Prior to blood collection, each participant underwent a comprehensive venepuncture assessment, which included an interview and a fasting questionnaire. The venepuncture protocols varied based on factors such as the participant's age, appointment type and session time. Specifically, the appropriate Nipro 19-, 21-, or 23-gauge × ¾ Butterfly Needle, along with the corresponding Nipro Luer adapter for use with the 19/21/23-gauge Saf-TEZ® set, were carefully selected depending on the subject's vein condition. The obtained specimens were securely stored in Vacutainer® tubes of suitable size and type and preserved under optimal frozen conditions (–30 °C) until their transportation to the University of Minnesota for analysis using the Cobas 6000 Chemistry Analyzer. All the procedures strictly adhered to the quality assurance and quality control (QA/QC) protocols of NHANES, in accordance with the standards outlined in the Clinical Laboratory Improvement Act of 1988.

### Selection of covariables

To explore the relationships between depression, the NHHR and infertility, potential confounding factors were adjusted based on the previous literature and clinically relevant variables. Potential covariables in the demographic data module included age (as continuous), race (categorized as Mexican American, other Hispanic, non-Hispanic black, non-Hispanic white, non-Hispanic Asian and other/multiracial), educational level (categorized as less than high school, high school and more than high school), marital status (categorized as married/living with partner, widowed/divorced/separated and never married) and poverty income ratio (PIR, as continuous). All these demographic data were directly accessible from the NHANES database without necessitating any form of transformation or manipulation, which ensured the integrity and fidelity of the data.

Additionally, covariables in the dietary data module included dietary cholesterol intake (as continuous) and caloric intake (as continuous). These data were determined using the means of two 24-h retrospective surveys that provided comprehensive data on nutrient consumption, including total nutrient intake and total dietary supplements. BMI covariables were derived from the examination data module and categorized into four levels: underweight (< 18.5 kg/m^2^), normal weight (18.5 to < 25 kg/m^2^), overweight (25 to < 30 kg/m^2^) and obese (≥ 30 kg/m^2^).

Furthermore, potential covariables from the laboratory data module encompassed serum levels of cotinine, vitamin D, aspartate aminotransferase (AST), alanine aminotransferase (ALT), triglyceride (TG), uric acid (UA) and creatinine (Cr). These variables were considered continuous. The covariables in the questionnaire data module contained drinking status (categorized as never, former and current drinker), smoking status (categorized as never, former and current smoker), history of pelvic infection (categorized as yes and no), regular periods (categorized as yes and no), sleeplessness (categorized as yes and no), history of diabetes (categorized as yes and no), history of hypertension (categorized as yes and no), physical activity (categorized as insufficient and sufficient) and sedentary behaviour (categorized as low sedentary time and high sedentary time).

The classification of history of pelvic infection, regular periods and sleeplessness can be directly divided according to the answer "yes" or "no". Participants' drinking status was ascertained through two pivotal inquiries: "Have you consumed at least 12 alcohol drinks in the past year?" and "Have you had at least 12 alcohol drinks in your lifetime?" Based on their responses, individuals were stratified as follows: those qualifying as current drinkers had consumed a minimum of 12 alcoholic drinks within the past year; former drinkers were defined as someone who had imbibed at least 12 alcoholic beverages in their lifetime but fewer than 12 drinks in the previous year; while those not meeting these criteria were categorized as never drinkers. Smoking status was assessed through two vital inquiries: "Have you smoked at least 100 cigarettes in your lifetime?" and "Do you currently smoke cigarettes?" Former smokers were classified as individuals who had smoked at least 100 cigarettes in their lifetime but were not presently smoking, while current smokers were those who were presently smoking. Individuals not meeting these criteria were categorized as never smokers. The history of diabetes was delineated as either receiving a diagnosis from a medical professional or utilizing insulin or diabetic pills to regulate blood sugar levels. Only those who met any of these conditions were considered to have diabetes. The history of hypertension was classified based on whether an individual had been diagnosed with hypertension by a doctor or was taking prescribed medications for hypertension. Only those who met any of these conditions were considered to have a history of hypertension.

In addition, the calculation of physical activity was derived from the metabolic equivalent (MET) scores indicated by the NHANES and the duration of the corresponding activity in a week (min). Individuals who were categorized as sufficiently active were those who met or above the cut-off point of 450 MET·min/week, while those who fell below this barrier were considered insufficiently active [23]. Sedentary behaviour was defined based on self-reported sedentary time, with a threshold of 6 h per day [23].

### Statistical analysis

The statistical analysis was performed in accordance with the protocols specified in the NHANES, which take into account the complexity of multistage cluster surveys and incorporate the weighting of pertinent samples. Depending on the nature of the data, continuous variables were evaluated using either the weighted Student's t test or the Wilcoxon rank-sum test, and the outcomes are reported as the weighted mean (SE) ± standard deviation (SD). Categorical variables were analysed with the weighted chi-square test with Rao & Scott's second-order correction, and the results are represented as the unweighted number of cases (n) and percentage (%).

To examine the associations between depression and infertility, as well as between the NHHR and infertility, a multivariable logistic regression analysis was employed with the calculation of odds ratios (ORs) and 95% confidence intervals (CIs). In addition, a multivariable linear regression analysis was also utilized to explore the relationship between depression states and the NHHR, with the calculation of β and 95% CIs. Three different models were used for these analyses: the crude model (without any adjustment for covariables), Model 1 (with adjustment for age, BMI and race) and Model 2 (with further adjustment for educational level, PIR, marital status, smoking status, sleeplessness, drinking status, history of pelvic infection, regular periods, physical activity, sedentary behaviour, dietary cholesterol, calories, serum cotinine, serum vitamin D, history of diabetes, history of hypertension, creatinine, ALT, AST, TG and UA). The data types for these covariables were consistent with those described in "Selection of covariables" section.

In addition, to further elucidate whether the NHHR levels mediated the connection between depression states and female infertility, mediation analysis was also carried out. The total effect denotes the direct association between depression states and female infertility, independent of the mediating variable (the NHHR). The mediation effect (ME) refers to the impact of depression on infertility through the NHHR, while the direct effect denotes the effect of depression on infertility after for the management of the NHHR. A significant ME suggests the existence of a mediating effect. In addition, subgroup analyses that take age and BMI into account were utilized to further explore the association between depression and infertility. Age categorization was defined by the tertiles of the continuous data.

The statistical analyses in this study were carried out with R (version 4.2.2, available at http://www.R-project.org). In particular, the "car" package was utilized to estimate multicollinearity, with variance inflation factors (VIFs) greater than 2 deemed suggestive of potential multicollinearity. In the present study, all the VIFs were less than 2, ruling out multicollinearity. Statistical significance was defined as a two-sided *P* value less than 0.05.

## Results

### Baseline characteristics of the participants

A total of 2,668 women meeting the screening criteria were ultimately enrolled in this study, comprising 305 infertility patients (representing a population of 4,887,069 women in the U.S.) and 2,363 controls (representing a population of 37,299,564 women in the U.S.), with weighted average ages of 34 ± 7 years and 31 ± 7 years, respectively (*P* < 0.001). In addition, there was no significant difference in the prevalence of infertility and depression across different age groups (Table S1). Notably, the prevalence rates of three different depression states in the infertility group were 64%, 20% and 16%, respectively. In comparison, the control group exhibited rates of 73%, 17% and 10% respectively. The difference between the two groups was statistically significant(*P* = 0.040). Additionally, the infertility group exhibited notably elevated NHHR levels in comparison to the control (*P* < 0.001). Furthermore, notable distinctions between the two groups were noted concerning BMI, marital status, history of pelvic infection, depression states, sleeplessness, history of diabetes, history of hypertension, the NHHR, HDL-C, ALT, TG and UA levels. However, no significant disparities were noted in serum vitamin D, cotinine, TC, AST, Cr, or dietary cholesterol levels or in caloric intake between the infertile population and controls. Table 1 illustrates all of the research population's detailed baseline characteristics.

**Table 1:** Baseline characteristics of the included participants

Characteristic	Overall (*N* = 42,186,633)	Fertility (*N* = 37,299,564)	Infertility (*N* = 4,887,069)	*P* value
**Age (years)**	32 ± 7	31 ± 7	34 ± 7	** < 0.001**
**Race**				0.708
Mexican American	475 (13%)	422 (13%)	53 (14%)	
Other Hispanic	280 (8.1%)	257 (8.4%)	23 (5.9%)	
Non-Hispanic White	876 (55%)	768 (55%)	108 (55%)	
Non-Hispanic Black	561 (13%)	491 (13%)	70 (14%)	
Non-Hispanic Asian	348 (6.8%)	312 (7.0%)	36 (5.8%)	
Other/multiracial	128 (4.4%)	113 (4.2%)	15 (5.7%)	
**BMI**				** < 0.001**
Underweight (< 18.5 kg/m^2^)	66 (2.3%)	60 (2.3%)	6 (1.8%)	
Normal weight (18.5 to < 25 kg/m^2^)	861 (34%)	783 (36%)	78 (24%)	
Overweight (25 to < 30 kg/m^2^)	642 (24%)	589 (25%)	53 (18%)	
Obese (≥ 30 kg/m^2^)	1,088 (39%)	920 (37%)	168 (56%)	
**Educational level**				0.460
Less than high school	409 (11%)	360 (11%)	49 (13%)	
High school	507 (20%)	447 (20%)	60 (20%)	
More than high school	1,752 (69%)	1,556 (69%)	196 (66%)	
**Marital status**				** < 0.001**
Married/Living with partner	1,520 (58%)	1,305 (56%)	215 (74%)	
Widowed/Divorced/Separated	275 (10%)	236 (9.6%)	39 (13%)	
Never married	873 (32%)	822 (35%)	51 (13%)	
**PIR**	2.63 ± 1.65	2.62 ± 1.65	2.68 ± 1.61	0.459
**Drinking status**				0.264
Never drinker	1,664 (60%)	1,463 (60%)	201 (63%)	
Former drinker	606 (20%)	530 (20%)	76 (22%)	
Current drinker	398 (19%)	370 (20%)	28 (15%)	
**Smoking status**				0.641
Never smoker	1,903 (68%)	1,700 (69%)	203 (65%)	
Former smoker	283 (12%)	245 (12%)	38 (13%)	
Current smoker	482 (19%)	418 (19%)	64 (21%)	
**History of pelvic infection**				**0.002**
Yes	132 (4.8%)	102 (4.0%)	30 (10%)	
No	2,519 (95%)	2,247 (96%)	272 (90%)	
**Regular periods**				0.359
Yes	2,514 (94%)	2,224 (95%)	290 (93%)	
No	154 (5.6%)	139 (5.4%)	15 (7.2%)	
**Depression states**				**0.040**
No depression	1,935 (72%)	1,740 (73%)	195 (64%)	
Minimal-to-mild depression	466 (17%)	401 (17%)	65 (20%)	
Moderate-to-severe depression	267 (11%)	222 (10%)	45 (16%)	
**Sleeplessness**				** < 0.001**
Yes	593 (24%)	495 (22%)	98 (36%)	
No	2,075 (76%)	1,868 (78%)	207 (64%)	
**History of diabetes**				** < 0.001**
Yes	162 (4.9%)	129 (4.0%)	33 (11%)	
No	2,506 (95%)	2,234 (96%)	272 (89%)	
**History of hypertension**				**0.010**
Yes	382 (12%)	322 (11%)	60 (17%)	
No	2,286 (88%)	2,041 (89%)	245 (83%)	
**Serum cotinine (ng/mL)**	41 ± 98	39 ± 96	49 ± 109	0.534
**Serum vitamin D (nmol/L)**	64 ± 26	64 ± 26	63 ± 25	0.709
**NHHR**	2.38 ± 1.17	2.34 ± 1.16	2.68 ± 1.25	** < 0.001**
**Physical activity**				0.221
Insufficient	796 (25%)	695 (24%)	101 (28%)	
Sufficient	1,872 (75%)	1,668 (76%)	204 (72%)	
**Sedentary behaviour**				0.828
Low sedentary time	1,286 (48%)	1,145 (48%)	141 (48%)	
High sedentary time	1,382 (52%)	1,218 (52%)	164 (52%)	
**Dietary cholesterol (mg)**	263 ± 169	263 ± 168	265 ± 178	0.754
**Dietary calories (kcal)**	1,859 ± 658	1,856 ± 651	1,877 ± 711	0.754
**TC (mg/dL)**	179 ± 35	179 ± 34	184 ± 35	0.088
**HDL-C (mg/dL)**	57 ± 15	57 ± 15	54 ± 16	**0.006**
**ALT (U/L)**	20 ± 16	19 ± 16	21 ± 11	**0.002**
**AST (U/L)**	21 ± 13	21 ± 13	21 ± 12	0.543
**TG (mmol/L)**	1.32 ± 1.37	1.29 ± 1.41	1.53 ± 1.03	** < 0.001**
**UA (umol/L)**	271 ± 66	269 ± 65	287 ± 67	** < 0.001**
**Cr (ng/mL)**	64 ± 17	64 ± 18	64 ± 12	0.868

*Abbreviations*: *BMI* body mass index, *PIR* poverty income ratio, *NHHR* non-HDL-cholesterol to HDL-cholesterol ratio, *TC* total cholesterol, *HDL-C* high-density lipoprotein cholesterol, *ALT* alanine aminotransferase, *AST* aspartate aminotransferase, *TG* triglyceride, *UA* uric acid, *Cr* creatinine

*P* in bold indicates a statistically significant difference

### Association between depression states and infertility

The study findings showed that, in comparison to individuals without depression, there was a substantial positive relationship between moderate-to-severe depression and a higher risk of experiencing infertile. This association was initially observed in the crude model and sustained to be statistically significant in the subsequent Model 1 and Model 2 with partial and complete adjustments, respectively, as indicated in Table 2. By adjusting for all covariables (Model 2), it was noted that individuals with moderate-to-severe depression states have a significantly higher likelihood, 1.98 times, of experiencing infertility compared to individuals without depression (OR_moderate-to-severe_: 1.98; 95% CI_moderate-to-severe_: 1.07–3.67; *P* = 0.03).

**Table 2:** The association between depression states and the risk of infertility

Depression	**Crude model**		**Model 1**		**Model 2**	
	**OR (95% CI)**	***P* value**	**OR (95% CI)**	***P* value**	**OR (95% CI)**	***P* value**
No depression	Ref		Ref		Ref	
Minimal-to-mild depression	1.36(0.86, 2.14)	0.18	1.33(0.83, 2.13)	0.22	1.36(0.81, 2.29)	0.21
Moderate-to-severe depression	1.86(1.08, 3.21)	**0.03**	1.81(1.03, 3.16)	**0.04**	1.98(1.07, 3.67)	**0.03**

Crude model: not adjusted for any covariables

Model 1: adjusted for age, BMI and race

Model 2: adjusted for age, BMI, race, educational level, marital status, PIR, smoking status, drinking status, sleeplessness, history of pelvic infection, regular periods, physical activity, sedentary behaviour, dietary cholesterol, calories, serum cotinine, serum vitamin D, history of diabetes, history of hypertension, creatinine, ALT, AST, TG and UA

*Abbreviations*: *OR* odds ratio, *CI* confidence interval, *Ref* reference

*P* in bold indicates a statistically significant difference

### Subgroup analysis based on age and BMI

In order to delve deeper into the connection between depression states and infertility, stratification analyses were carried out based on age and BMI subgroup, and weighted logistic regression was applied to evaluate the association between depression states and the odds of infertility. Notably, there was a significant difference in the proportion of patients with varying degrees of depression in the 18–28 and 37–45 age subgroups (Table S2). Specifically, a greater proportion of individuals with minimal-to-mild and moderate-to-severe depression was observed among the infertility population, while no such difference was found in the 29–36 age subgroup. Additionally, the mean NHHR was greater in the 18–28 and 29–36 subgroups of infertile individuals (18–28 subgroup: fertility: 2.14 ± 1.03, infertility: 2.82 ± 1.46, *P* = 0.001; 29–36 subgroup: fertility: 2.40 ± 1.13, infertility: 2.73 ± 1.21, *P* = 0.035), but there was no statistically significant difference in the 37–45 age subgroup (*P* > 0.05). The weighted logistic regression analysis indicated that in the 18–28 subgroup, individuals with moderate-to-severe depression had 1.27 times greater odds of infertility than those without depression tendencies (Model 2: OR_moderate-to-severe_: 2.27; 95% CI_moderate-to-severe_: 1.06–4.86, *P* = 0.036; Table 3). In the 37–45 subgroup, the odds of infertility increased by 1.15 and 1.85 times in individuals with minimal-to-mild and moderate-to-severe depression, respectively (Model 2: OR_minimal-to-mild_: 2.15; 95% CI_minimal-to-mild_: 1.07–4.33, *P* = 0.034; OR_moderate-to-severe_: 2.85; 95% CI_moderate-to-severe_: 1.02, 8.01, *P* = 0.047). No significant association between various depression states and female infertility was found within the 29–36 subgroup (*P* > 0.05). According to the BMI subgroup analysis using thresholds of 25 and 30, there was no significant difference in the depression's prevalence among the underweight/normal weight, overweight or obese groups (Table S3). In addition, the NHHR did not significantly differ among these three subgroups. The results from weighted logistic regression analysis revealed that moderate-to-severe depression was positively associated with infertility solely in the population with a BMI ≥ 30 kg/m^2^, with a 0.92-fold increase in the odds of infertility (OR_moderate-to-severe_: 1.92, 95% CI_moderate-to-severe_: 1.01–3.65, *P* = 0.048; Table 4).

**Table 3:** The association of depression states with infertility in different age subgroups

	**Crude model**		**Model 1**		**Model 2**	
	**OR (95% CI)**	***P* value**	**OR (95% CI)**	***P* value**	**OR (95% CI)**	***P* value**
**18–28 years**						
No depression	Ref		Ref		Ref	
Minimal-to-mild depression	2.18 (0.99, 4.82)	0.054	1.93 (0.79, 4.70)	0.142	2.19 (0.92, 5.21)	0.072
Moderate-to-severe depression	2.53 (1.02, 6.30)	**0.046**	2.51 (0.94, 6.73)	0.066	2.27 (1.06, 4.86)	**0.036**
**29–36 years**						
No depression	Ref		Ref		Ref	
Minimal-to-mild depression	0.85 (0.43, 1.67)	0.623	0.86 (0.43, 1.74)	0.668	0.72 (0.33, 1.57)	0.379
Moderate-to-severe depression	0.90 (0.29, 2.79)	0.849	0.76 (0.22, 2.60)	0.652	0.68 (0.23, 2.05)	0.469
**37–45 years**						
No depression	Ref		Ref		Ref	
Minimal-to-mild depression	1.88 (0.98, 3.60)	0.059	1.95 (1.02, 3.72)	**0.044**	2.15 (1.07, 4.33)	**0.034**
Moderate-to-severe depression	3.00 (1.28, 7.04)	**0.013**	2.93 (1.20, 7.15)	**0.020**	2.85(1.02, 8.01)	**0.047**

Crude model: not adjusted for any covariables

Model 1: adjusted for BMI and race

Model 2: adjusted for BMI, race, educational level, marital status, PIR, smoking status, drinking status, sleeplessness, history of pelvic infection, regular periods, physical activity, sedentary behaviour, dietary cholesterol, calories, serum cotinine, serum vitamin D, history of diabetes, history of hypertension, creatinine, ALT, AST, TG and UA

*P* in bold indicates a statistically significant difference

**Table 4:** The association of depression states with infertility in different BMI subgroups

	**Crude model**		**Model 1**		**Model 2**	
	**OR (95% CI)**	***P* value**	**OR (95% CI)**	***P* value**	**OR (95% CI)**	***P* value**
**< 25 kg/m**^**2**^						
No depression	Ref		Ref		Ref	
Minimal-to-mild depression	1.10 (0.42, 2.85)	0.847	1.22 (0.46, 3.21)	0.685	1.12 (0.35, 3.57)	0.843
Moderate-to-severe depression	2.33 (0.86, 6.31)	0.094	2.58 (0.97, 6.81)	0.056	2.54 (0.80, 8.06)	0.107
**25 to < 30 kg/m**^**2**^						
No depression	Ref		Ref		Ref	
Minimal-to-mild depression	1.12 (0.39, 3.24)	0.833	1.21 (0.35, 4.17)	0.753	1.18 (0.36, 3.84)	0.772
Moderate-to-severe depression	1.68 (0.43, 6.63)	0.447	1.84 (0.53, 6.45)	0.330	2.26 (0.62, 8.27)	0.203
** ≥ 30 kg/m**^**2**^						
No depression	Ref		Ref		Ref	
Minimal-to-mild depression	1.39 (0.76, 2.57)	0.281	1.43 (0.76, 2.68)	0.254	1.48 (0.78, 2.81)	0.214
Moderate-to-severe depression	1.57 (0.84, 2.94)	0.155	1.66 (0.86, 3.18)	0.126	1.92 (1.01, 3.65)	**0.048**

Crude model: not adjusted for any covariables

Model 1: adjusted for age and race

Model 2: adjusted for age, race, educational level, marital status, PIR, smoking status, drinking status, sleeplessness, history of pelvic infection, regular periods, physical activity, sedentary behaviour, dietary cholesterol, calories, serum cotinine, serum vitamin D, history of diabetes, history of hypertension, creatinine, ALT, AST, TG and UA

*P* in bold indicates a statistically significant difference

### Association between the NHHR and infertility

Table 5 elaborates on the outcomes of the weighted multivariable logistic regression analysis, highlighting that the NHHR levels were positively associated with the occurrence of infertility across various models. When the NHHR was considered a continuous variable, the results showed that each incremental unit rise in the NHHR was associated with a noteworthy 0.18-fold increase in the odds of infertility (Model 2: OR_NHHR_: 1.18, 95% CI_NHHR_: 1.01–1.39, *P* = 0.044). In order to gain deeper insights into this association, the NHHR was further categorized into discrete intervals (quartiles), revealing that individuals who fell within the third and fourth quartiles exhibited a significantly greater likelihood of experiencing infertility than did those in the first quartile (Model 2: Q3: OR_NHHR_: 1.93, 95% CI_NHHR_: 1.15–3.25, *P* = 0.018; Q4: OR_NHHR_: 1.90, 95% CI_NHHR_: 1.06–3.41, *P* = 0.034).

**Table 5:** The association between the NHHR and the risk of infertility

	**Crude model**		**Model 1**		**Model 2**	
	**OR (95% CI)**	***P* value**	**OR (95% CI)**	***P* value**	**OR (95% CI)**	***P* value**
NHHR	1.24(1.10, 1.39)	** < 0.001**	1.19 (1.06, 1.35)	**0.005**	1.18(1.01, 1.39)	**0.044**
NHHR quartiles						
Quartile 1	Ref		Ref		Ref	
Quartile 2	1.42(0.89, 2.28)	0.139	1.42(0.88, 2.30)	0.150	1.53(0.87, 2.71)	0.126
Quartile 3	2.02(1.23, 3.32)	**0.006**	1.89(1.15, 3.11)	**0.014**	1.93(1.15, 3.25)	**0.018**
Quartile 4	2.42(1.46, 4.03)	**0.001**	2.08(1.24, 3.49)	**0.007**	1.90(1.06, 3.41)	**0.034**

Quartile 1: 0.4017094–1.563636; Quartile 2: 1.565217–2.146341; Quartile 3: 2.148148–2.921569; Quartile 4: 2.921875–13.926829

### Association between depression states and the NHHR

Table 6 delineates the associations between depression states and the NHHR. The weighted multivariable linear regression results revealed a significant positive relationship between moderate-to-severe depression and the NHHR in the crude model (β: 0.22, 95% CI: 0.03–0.40; *P* = 0.024). Prominently, this association remained significant even after accounting for the adjustment for partial and all covariables.

**Table 6:** The association between depression states and the NHHR

Depression	**Crude model**		**Model 1**		**Model 2**	
	**β (95% CI)**	***P* value**	**β (95% CI)**	***P* value**	**β (95% CI)**	***P* value**
No depression	Ref		Ref		Ref	
Minimal-to-mild depression	0.06(-0.10, 0.22)	0.459	0.06(-0.09, 0.22)	0.405	0.05(-0.11, 0.21)	0.512
Moderate-to-severe depression	0.22(0.03, 0.40)	**0.024**	0.25(0.07, 0.42)	**0.008**	0.20(0.01, 0.39)	**0.043**

Crude model: not adjusted for any covariables

Model 1: adjusted for age, BMI and race

Model 2: adjusted for age, BMI, race, educational level, marital status, PIR, smoking status, drinking status, sleeplessness, history of pelvic infection, regular periods, physical activity, sedentary behaviour, dietary cholesterol, calories, serum cotinine, serum vitamin D, history of diabetes, history of hypertension, creatinine, ALT, AST, TG and UA

*P* in bold indicates a statistically significant difference

### Mediating effect of the NHHR on depression states and the risk of infertility

**Fig. 2:**
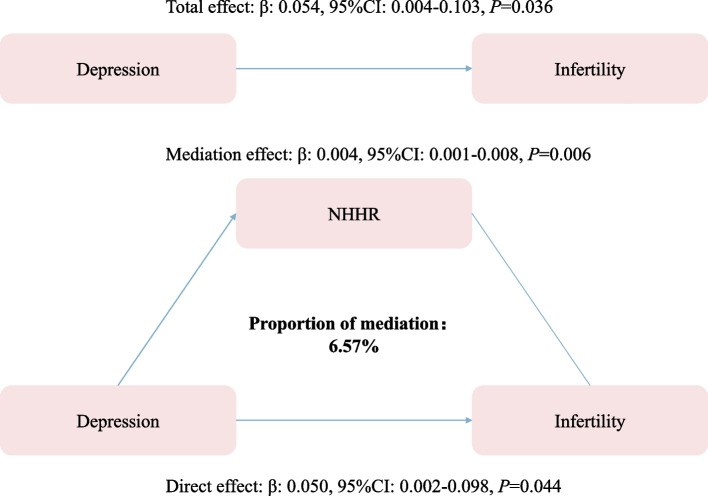
Mediation analysis of the NHHR on the risk of depression and infertility

Mediation analysis was performed to further assess the mediating effect of the NHHR on the risk of depression and infertility. As shown in Fig. 2, the depression states exhibited a significant association with infertility risk (total effect: 0.054, 95% CI: 0.004–0.103, *P* = 0.036), and the NHHR had a significant mediating effect on the depression state and infertility (mediation effect: 0.004, 95% CI: 0.001–0.008, *P* = 0.006), explaining 6.57% of this association.

## Discussion

In this comprehensive study involving 2,668 women aged 18–45 years, the results showed that individuals exhibiting moderate-to-severe depression had an increased risk of experiencing infertility. Importantly, this relationship persisted as significant even after adjusting for demographic, socioeconomic, lifestyle habits, and past medical history. Subgroup analysis further revealed that while depression is positively linked to infertility risk within specific age subgroups, no such association was found within the subgroup categorized by BMI. In addition, this research has demonstrated that an elevated NHHR is a contributing factor to infertility, and that moderate-to-severe depression is positively correlated with the NHHR. Significantly, mediation analysis further revealed that the association between depression states and female infertility was partially mediated by the NHHR.

In 2008, the WHO identified major depressive disorder (MDD) as the third leading contributor to the global disease burden, with projections indicating that it will rank first by 2030 [24]. However, the coronavirus disease 2019 pandemic has accelerated this trajectory, causing a significant increase in the incidence of MDD, affecting approximately 53 million individuals—a 27.6% increase—most notably among women and younger people [25]. Stressful events, such as the pandemic, have notably impacted women, resulting in diminished sexual satisfaction, reduced desire for pregnancy, and increased incidence of menstrual disorders [26, 27]. Moreover, depression and anxiety have direct adverse effects on cyclical sex hormone secretion, endometrial growth, and the clinical pregnancy rate in infertile patients and are closely correlated with the success of infertility treatment [28, 29]. The outcomes of a Mendelian randomization (MR) study also supported the findings of this research [30]. These findings align with the outcomes of the present study, providing further evidence for the negative impact of depression on reproductive health.

Previous studies have emphasized the pivotal role of the hypothalamic‒pituitary‒adrenal (HPA) axis in the pathogenesis of depression, with aberrant activation leading to corticotropin-releasing hormone (CRH) release and elevated plasma cortisol [24, 31]. An increase in endogenous CRH has been found to suppress gonadotropin secretion and hinder the synthesis and secretion of oestradiol in both rat and human granulosa cells, suggesting that CRH may directly interfere with the function of the HPA axis, particularly in relation to the ovaries [32, 33]. Elevated serum cortisol in infertile women is positively correlated with anxiety and depression severity, while increased cortisol and prolactin levels negatively affect oestradiol secretion, thereby disrupting the menstrual cycle and fertility [28, 34]. In addition, cortisol was shown to impair the developmental potential of oocytes and trigger apoptosis in mural granulosa cells and cumulus cells, thereby exacerbating reproductive challenges [35, 36]. These findings collectively underscore depression's detrimental impact on the female reproductive system, potentially contributing to infertility, further supporting this study's conclusions.

As a newly emerging indicator, the NHHR has garnered extensive attention in recent years for its role in atherosclerosis, diabetes and other diseases. Although conclusive evidence of a link between the NHHR and mental disorders, particularly depression, is lacking, multiple studies have investigated the intricate interplay between various types of cholesterol and depression. Enko et al. discovered that individuals who suffered from depression exhibited elevated levels of TG and lower levels of HDL-C [37]. Higher TC/HDL-C and LDL-C/HDL-C ratios were linked to accelerated depressive symptom escalation in females [38]. Similarly, Kuwano et al. identified decreased HDL-C levels as a potential depression severity biomarker in MDD patients, particularly in association with 'affective' subsymptoms such as the loss of pleasure and sadness [39]. Additionally, several cross-sectional studies based on the U.S. population have shown that both residual cholesterol and non-HDL-C levels are positively associated with depression [40, 41]. Conversely, Oh et al. identified a notable association between elevated HDL-C levels and depression in adult males, while higher TG levels were linked to depression in adult females [42]. These studies indicate a complex relationship between different cholesterol types and depression. A multitude of population-based studies are still warranted to explain the connection between cholesterol and depression across different sexes and among various subtypes of depression.

The association between cholesterol and infertility has been extensively explored. Verit et al. observed higher levels of TG, TC, LDL-C and high-sensitivity C-reactive protein (hs-CRP) in individuals with unexplained infertility, alongside decreased levels of HDL-C [43]. Additionally, TG, HDL-C and hs-CRP were identified as independent risk factors for unexplained infertility [43]. In contrast, an MR study revealed that the likelihood of female infertility was raised by 13% and 16% for every incremental rise in LDL-C and TC levels, respectively, and there was no evidence that HDL-C was prominently related to female infertility risk [44]. Notably, HDL is the primary lipoprotein present in ovarian follicular fluid (FF), which is mainly derived from plasma [45]. Previous studies have provided evidence supporting the detrimental effects of an imbalance in crosstalk between FF HDL and oocytes on the female reproductive system. A study on scavenger receptor class B type I knockout (SR-B1 KO) mice with hypercholesterolemia showed that cholesterol accumulation in oocytes starts from the antral follicular stage [45]. Knockout of ATP-binding cassette subfamily A member 1 (ABCA1) in a consistent genetic background hindered the efflux of cholesterol in the ovaries and resulted in an accumulation of cholesterol in oocytes, causing a distinct flattened and dark characteristic indicative of oocyte death [45]. These findings highlight the necessity of cholesterol efflux from oocytes to functional HDL, regulated by FF HDL and ABC transporters, to maintain optimal intracellular cholesterol levels within the physiological range and preserve female fertility.

Cholesterol in the body exists as free cholesterol (FC) and cholesteryl ester (CE). Trigatti et al. discovered that homozygous SR-B1 KO female mice were infertile and displayed diminished lipid staining in the corpus luteum of the ovary, indicating a decrease in CE storage [46]. Nonetheless, there were no significant abnormalities observed in the oestrous cycles or the number of ovulated eggs, suggesting that normal cholesterol storage is not crucial for the production of an adequate number of steroid hormones necessary for female fertility [46]. SR-B1 knockout mice are still able to maintain partial ovarian function by utilizing some cholesterol but may not be able to maintain sufficient steroid hormone production for pregnancy. Similarly, Liu et al. reported a decrease in TC, phospholipid and CE contents in the ovaries of SR-B1 knockout mice, while no significant alterations were noted in ovarian FC and TG levels [47]. However, an elevation in ovarian mol% FC and FC/TC ratio was observed [47], reflecting the transformation of intracellular FC to CE and a state of heightened bioavailability of HDL-FC to a certain extent. This discovery aligns with the observations of Rosales et al., who also demonstrated that the disruption of HDL structure using adeno-associated virus loaded with serum opacity factor effectively decreased the HDL-FC concentration [48]. This intervention not only restored ovarian size and corpus luteum formation in infertile mice but also led to a reduction in CE content and an elevation in the FC/TC ratio [48], which indicated that lowering the bioavailability of HDL plays a vital role in restoring fertility. Unsurprisingly, Miettinen et al. also observed that administering probucol or inactivating the *ApoA1* gene in SR-B1 KO mice ameliorated the abnormal structure and/or quantity of HDL and restored fertility [49]. These results indicate the presence of a well-regulated cholesterol homeostasis system within the ovary that controls the transportation of FF HDL and oocytes, with profound implications for female fertility.

## Study strengths and limitations

In summary, this research revealed the importance of the NHHR as an innovative biomarker in the field of depression and reproductive health. The NHHR makes full use of clinical information on non-HDL-C and HDL-C, which are easily obtained and convenient to calculate, and fully considers the effects of different types of cholesterol. In addition, this study introduces the NHHR as a mediator variable for the first time, creatively exploring its mediating function in the association between depression and infertility, which can contribute to the understanding of the pathophysiological mechanisms linking depression and infertility and provide valuable insights for early clinical diagnosis and intervention.

However, it is also necessary to acknowledge the limitations of this study. Firstly, due to the nature of the data collected from the NHANES, all relevant information in this study was available only from three cycles spanning from 2013 to 2018, and data on male infertility were not accessible, which may impose certain constraints to the overall conclusions. Secondly, the cross-sectional design of this study inherently restricts the capacity to determine a causal relationship between depression and infertility. Thirdly, the observation objects of this study were screened based on the nationwide random sampling of the NHANES rather than being specifically designed for the study of a particular population (in this case, the infertile population). This approach may introduce sample selection bias, as the study sample may not offer a comprehensive representation of the entire infertile population. While efforts were made to mitigate this bias with population weighting, the potential for bias remains. Fourth, factors such as the social stigma associated with infertility and reliance on recall interviews may introduce information and recall biases. Finally, despite comprehensive adjustments for various confounders, there may still exist unknown potential confounding factors that could influence the observed associations. Therefore, the results of this study should be interpreted cautiously and objectively.

## Conclusion

In conclusion, this study provides initial confirmation of the link between depression and heightened infertility risk, suggesting that comprehensive cholesterol levels represented by the NHHR may serve as one potential mechanism elucidating this association. The NHHR is expected to be an indicator of clinical depression-related infertility. However, to deepen the understanding of this relationship, future investigations should explore the impact of additional psychosocial factors on infertility, as well as the diagnostic utility of biomarkers in both the occurrence and progression of infertility. Furthermore, longitudinal studies are necessary to examine the evolution of these factors over time and their correlation with fertility outcomes. Clinicians are advised to prioritize monitoring the mental health status and NHHR values of women across various age groups, as addressing these factors may help mitigate the risk of infertility.

### Supplementary Information

Supplementary Material 1.

## Data Availability

No datasets were generated or analysed during the current study.
