# Supplementary material for: Association between depression and infertility risk among American women aged 18–45 years: the mediating effect of the NHHR
Source: Lipids Health Dis. 2024 Jun 10;23:178. doi: 10.1186/s12944-024-02164-3 (PMC11163758; doi:10.1186/s12944-024-02164-3)
Supplement: Supplementary file 1 — Supplementary Material 1. [file 12944_2024_2164_MOESM1_ESM.docx]

Table S1. Weighted prevalence of infertility and depression based on different age groups.

| **Characteristic** | **18-42 years**  **(N=38,873,338)** | **43-45 years**  **(N=3,313,295)** | ***P* value** |
| --- | --- | --- | --- |
| **Infertility** |  |  | 0.079 |
| Yes | 270 (11%) | 35 (16%) |  |
| No | 2,160(89%) | 203 (84%) |  |
| **Depression states** |  |  | 0.360 |
| No depression | 1,765 (72%) | 170 (70%) |  |
| Minimal-to-mild depression | 425 (17%) | 41 (15%) |  |
| Moderate-to-severe depression | 240 (11%) | 27 (15%) |  |

Table S2. Subgroup analysis based on age.

| **Characteristic** | **18-28 years** | | | **29-36 years** | | | **37-45 years** | | |
| --- | --- | --- | --- | --- | --- | --- | --- | --- | --- |
|  | **Fertility**  **(N=15,473,342)** | **Infertility**  **(N=1,011,057)** | ***p*-value** | **Fertility**  **(N=11,367,646)** | **Infertility**  **(N=1,964,459)** | ***p*-value** | **Fertility**  **(N=10,458,576)** | **Infertility (N=1,911,553)** | ***P* value** |
| **Race** |  |  | 0.247 |  |  | 0.454 |  |  | 0.225 |
| Mexican American | 148 (13%) | 20 (25%) |  | 117 (11%) | 15 (12%) |  | 157 (14%) | 18 (9.4%) |  |
| Other Hispanic | 99 (8.3%) | 4 (11%) |  | 71 (8.0%) | 5 (3.2%) |  | 87 (9.0%) | 14 (6.0%) |  |
| Non-Hispanic White | 319 (56%) | 19 (37%) |  | 229 (55%) | 48 (61%) |  | 220 (52%) | 41 (59%) |  |
| Non-Hispanic Black | 172 (13%) | 18 (16%) |  | 160 (13%) | 27 (16%) |  | 159 (13%) | 25 (11%) |  |
| Non-Hispanic Asian | 98 (6.5%) | 3 (4.0%) |  | 108 (7.1%) | 10 (5.0%) |  | 106 (7.6%) | 23 (7.5%) |  |
| Other/  multiracial | 42 (3.8%) | 1 (7.1%) |  | 45 (5.1%) | 4 (3.1%) |  | 26 (3.8%) | 10 (7.5%) |  |
| **BMI** |  |  | **0.035** |  |  | **0.001** |  |  | 0.849 |
| Underweight  (<18.5 kg/m^2^) | 39 (3.8%) | 3 (3.4%) |  | 12 (1.3%) | 2 (2.0%) |  | 9 (1.3%) | 1 (0.9%) |  |
| Normal weight  (18.5 to <25 kg/m^2^) | 349 (41%) | 18 (22%) |  | 221 (32%) | 19 (20%) |  | 213 (31%) | 41 (30%) |  |
| Overweight (25 to <30 kg/m^2^) | 201 (24%) | 9 (21%) |  | 208 (29%) | 21 (15%) |  | 180 (23%) | 23 (19%) |  |
| Obese  (≥30 kg/m^2^) | 289 (31%) | 35 (54%) |  | 287 (38%) | 67 (63%) |  | 344 (45%) | 66 (50%) |  |
| **Educational level** |  |  | **0.005** |  |  | 0.667 |  |  | 0.820 |
| Less than high school | 92 (8.0%) | 13 (16%) |  | 114 (11%) | 17 (12%) |  | 154 (14%) | 19 (12%) |  |
| High school | 196 (24%) | 26 (42%) |  | 116 (15%) | 12 (12%) |  | 135 (20%) | 22 (18%) |  |
| More than high school | 590 (68%) | 26 (41%) |  | 500 (74%) | 80 (76%) |  | 466 (66%) | 90 (70%) |  |
| **Marital status** |  |  | **0.008** |  |  | **0.032** |  |  | 0.193 |
| Married/ Living with partner | 352 (40%) | 40 (69%) |  | 440 (65%) | 81 (80%) |  | 513 (68%) | 94 (72%) |  |
| Widowed/ Divorced/ Separated | 32 (3.4%) | 2 (4.3%) |  | 80 (9.4%) | 11 (8.1%) |  | 124 (19%) | 26 (23%) |  |
| Never married | 494 (56%) | 23 (27%) |  | 210 (25%) | 17 (12%) |  | 118 (13%) | 11 (5.7%) |  |
| **PIR** | 2.40 ± 1.55 | 1.73 ± 1.12 | **0.002** | 2.80 ± 1.72 | 2.70 ± 1.56 | 0.651 | 2.74 ± 1.69 | 3.16 ± 1.67 | 0.080 |
| **Drinking status** |  |  | 0.628 |  |  | 0.503 |  |  | 0.133 |
| Never drinker | 558 (60%) | 43 (54%) |  | 457 (59%) | 66 (60%) |  | 448 (60%) | 92 (71%) |  |
| Former drinker | 165 (17%) | 16 (23%) |  | 161 (21%) | 30 (25%) |  | 204 (25%) | 30 (19%) |  |
| Current drinker | 155 (23%) | 6 (23%) |  | 112 (20%) | 13 (15%) |  | 103 (15%) | 9 (9.7%) |  |
| **Smoking status** |  |  | **0.022** |  |  | 0.255 |  |  | 0.552 |
| Never smoker | 671 (74%) | 41 (59%) |  | 505 (68%) | 73 (65%) |  | 524 (63%) | 89 (69%) |  |
| Former smoker | 63 (7.6%) | 4 (2.9%) |  | 82 (13%) | 17 (19%) |  | 100 (17%) | 17 (12%) |  |
| Current smoker | 144 (19%) | 20 (38%) |  | 143 (19%) | 19 (16%) |  | 131 (20%) | 25 (18%) |  |
| **History of pelvic infection** |  |  | **0.004** |  |  | 0.176 |  |  | 0.145 |
| Yes | 25 (3.1%) | 6 (14%) |  | 35 (4.1%) | 10 (8.0%) |  | 42 (5.4%) | 14 (11%) |  |
| No | 849 (97%) | 58 (86%) |  | 691 (96%) | 98 (92%) |  | 707 (95%) | 116 (89%) |  |
| **Regular periods** |  |  | **0.037** |  |  | 0.891 |  |  | 0.949 |
| Yes | 833 (95%) | 59 (88%) |  | 685 (96%) | 104 (96%) |  | 706 (92%) | 127 (92%) |  |
| No | 45 (4.6%) | 6 (12%) |  | 45 (4.1%) | 5 (4.4%) |  | 49 (8.0%) | 4 (7.7%) |  |
| **Depression states** |  |  | **0.037** |  |  | 0.880 |  |  | **0.011** |
| No depression | 631 (70%) | 32 (50%) |  | 554 (75%) | 80 (78%) |  | 555 (75%) | 83 (56%) |  |
| Minimal-to-mild depression | 165 (18%) | 18 (28%) |  | 107 (17%) | 19 (15%) |  | 129 (14%) | 28 (20%) |  |
| Moderate-to-severe depression | 82 (12%) | 15 (21%) |  | 69 (7.8%) | 10 (7.2%) |  | 71 (10%) | 20 (23%) |  |
| **Sleeplessness** |  |  | 0.440 |  |  | 0.073 |  |  | **0.006** |
| Yes | 149 (19%) | 14 (25%) |  | 153 (23%) | 34 (33%) |  | 193 (27%) | 50 (45%) |  |
| No | 729 (81%) | 51 (75%) |  | 577 (77%) | 75 (67%) |  | 562 (73%) | 81 (55%) |  |
| **History of diabetes** |  |  | **0.005** |  |  | **0.001** |  |  | 0.093 |
| Yes | 18 (2.0%) | 6 (7.8%) |  | 35 (3.5%) | 11 (12%) |  | 76 (7.5%) | 16 (12%) |  |
| No | 860 (98%) | 59 (92%) |  | 695 (96%) | 98 (88%) |  | 679 (92%) | 115 (88%) |  |
| **History of hypertension** |  |  | 0.609 |  |  | 0.282 |  |  | 0.214 |
| Yes | 63 (5.8%) | 7 (7.2%) |  | 98 (12%) | 20 (17%) |  | 161 (18%) | 33 (24%) |  |
| No | 815 (94%) | 58 (93%) |  | 632 (88%) | 89 (83%) |  | 594 (82%) | 98 (76%) |  |
| **Serum cotinine (ng/mL)** | 33 ± 80 | 70 ± 100 | **0.021** | 41 ± 102 | 42 ± 111 | 0.354 | 48 ± 110 | 47 ± 110 | 0.500 |
| **Serum vitamin D (nmol/L)** | 63 ± 24 | 55 ± 21 | 0.083 | 64 ± 26 | 62 ± 22 | 0.747 | 66 ± 26 | 70 ± 28 | 0.494 |
| **NHHR** | 2.14 ± 1.03 | 2.82 ± 1.46 | **0.001** | 2.40 ± 1.13 | 2.73 ± 1.21 | **0.035** | 2.55 ± 1.31 | 2.56 ± 1.17 | 0.627 |
| **Physical activity** |  |  | 0.517 |  |  | 0.680 |  |  | 0.666 |
| Insufficient | 217 (22%) | 21 (26%) |  | 205 (22%) | 33 (24%) |  | 273 (30%) | 47 (32%) |  |
| Sufficient | 661 (78%) | 44 (74%) |  | 525 (78%) | 76 (76%) |  | 482 (70%) | 84 (68%) |  |
| **Sedentary behavior** |  |  | 0.363 |  |  | 0.702 |  |  | 0.961 |
| Low sedentary time | 400 (47%) | 32 (54%) |  | 353 (47%) | 48 (44%) |  | 392 (50%) | 61 (50%) |  |
| High sedentary time | 478 (53%) | 33 (46%) |  | 377 (53%) | 61 (56%) |  | 363 (50%) | 70 (50%) |  |
| **Dietary cholesterol (mg)** | 263 ± 166 | 249 ± 144 | 0.860 | 269 ± 171 | 271 ± 184 | 0.720 | 257 ± 168 | 267 ± 187 | 0.988 |
| **Dietary calories (kcal)** | 1,852 ± 628 | 1,872 ± 727 | 0.984 | 1,859 ± 709 | 1,940 ± 735 | 0.270 | 1,861 ± 618 | 1,815 ± 679 | 0.605 |
| **TC (mg/dL)** | 169 ± 32 | 177 ± 37 | 0.492 | 183 ± 33 | 184 ± 37 | 0.878 | 189 ± 36 | 187 ± 33 | 0.830 |
| **HDL-C (mg/dL)** | 57 ± 14 | 50 ± 13 | **0.001** | 57 ± 15 | 52 ± 14 | **0.029** | 57 ± 17 | 57 ± 18 | 0.595 |
| **ALT (U/L)** | 19 ± 13 | 22 ± 14 | **0.019** | 20 ± 15 | 20 ± 10 | 0.246 | 20 ± 21 | 21 ± 10 | **0.015** |
| **AST (U/L)** | 20.4 ± 8.0 | 20.5 ± 7.0 | 0.983 | 22 ± 11 | 20 ± 7 | 0.233 | 22 ± 19 | 24 ± 17 | **0.045** |
| **TG (mmol/L)** | 1.14 ± 0.66 | 1.55 ± 1.20 | **<0.001** | 1.27 ± 0.79 | 1.53 ± 0.81 | **0.022** | 1.53 ± 2.38 | 1.52 ± 1.13 | 0.905 |
| **UA (umol/L)** | 273 ± 63 | 293 ± 73 | 0.160 | 269 ± 65 | 285 ± 73 | 0.150 | 262 ± 69 | 285 ± 58 | **<0.001** |
| **Cr (ng/mL)** | 64 ± 21 | 62 ± 13 | 0.586 | 64 ± 12 | 64 ± 12 | 0.759 | 65 ± 18 | 63 ± 11 | 0.914 |

Abbreviations: BMI: body mass index; PIR, poverty income ratio; NHHR: non-HDL-cholesterol to HDL-cholesterol ratio; TC: total cholesterol; HDL-C: high-density lipoprotein cholesterol; ALT: alanine aminotransferase; AST: aspartate aminotransferase; TG: triglyceride; UA: uric acid; Cr: creatinine. P in bold indicates a significant statistical difference. *P* in bold indicates a significant statistical difference.

Table S3. Subgroup analysis based on BMI.

| **Characteristic** | **BMI < 25 kg/m^2^** | | | **25 ≤ BMI < 30 kg/m^2^** | | | **BMI ≥ 30 kg/m^2^** | | |
| --- | --- | --- | --- | --- | --- | --- | --- | --- | --- |
|  | **Fertility**  **(N=14,103,862)** | **Infertility**  **(N=1,277,463)** | ***P*-value** | **Fertility**  **(N=9,346,228)** | **Infertility**  **(N=873,400)** | ***P*-value** | **Fertility**  **(N=13,849,474)** | **Infertility**  **(N=2,736,206)** | ***P* value** |
| **Age(years)** | 30 ± 7 | 35 ± 7 | **<0.001** | 31 ± 7 | 35 ± 7 | **0.009** | 32 ± 7 | 34 ± 6 | **0.020** |
| **Race** |  |  | 0.715 |  |  | 0.378 |  |  | 0.149 |
| Mexican American | 80 (7.9%) | 8 (4.3%) |  | 142 (15%) | 10 (12%) |  | 200 (16%) | 35 (19%) |  |
| Other Hispanic | 95 (8.4%) | 9 (6.7%) |  | 67 (8.4%) | 4 (11%) |  | 95 (8.4%) | 10 (3.8%) |  |
| Non-Hispanic White | 308 (61%) | 29 (64%) |  | 187 (56%) | 17 (43%) |  | 273 (47%) | 62 (55%) |  |
| Non-Hispanic Black | 121 (8.2%) | 15 (11%) |  | 103 (9.9%) | 10 (15%) |  | 267 (20%) | 45 (15%) |  |
| Non-Hispanic Asian | 200 (12%) | 21 (13%) |  | 68 (5.9%) | 9 (7.5%) |  | 44 (2.9%) | 6 (1.7%) |  |
| Other/ multiracial | 39 (3.2%) | 2 (1.7%) |  | 22 (3.9%) | 3 (12%) |  | 52 (5.4%) | 10 (5.7%) |  |
| **Educational level** |  |  | 0.572 |  |  | 0.125 |  |  | 0.849 |
| Less than high school | 88 (7.7%) | 12 (11%) |  | 111 (11%) | 9 (11%) |  | 161 (13%) | 28 (15%) |  |
| High school | 124 (16%) | 16 (20%) |  | 113 (21%) | 6 (9.5%) |  | 210 (24%) | 38 (24%) |  |
| More than high school | 631 (77%) | 56 (69%) |  | 365 (68%) | 38 (80%) |  | 560 (62%) | 102 (61%) |  |
| **Marital status** |  |  | **<0.001** |  |  | 0.330 |  |  | **<0.001** |
| Married/ Living with partner | 453 (54%) | 58 (77%) |  | 359 (62%) | 36 (71%) |  | 493 (53%) | 121 (74%) |  |
| Widowed/ Divorced/ Separated | 59 (5.3%) | 11 (7.6%) |  | 64 (13%) | 6 (16%) |  | 113 (12%) | 22 (14%) |  |
| Never married | 331 (41%) | 15 (16%) |  | 166 (25%) | 11 (12%) |  | 325 (35%) | 25 (11%) |  |
| **PIR** | 2.89 ± 1.68 | 2.77 ± 1.63 | 0.600 | 2.63 ± 1.68 | 3.26 ± 1.58 | 0.068 | 2.33 ± 1.56 | 2.46 ± 1.57 | 0.205 |
| **Drinking status** |  |  | 0.346 |  |  | 0.593 |  |  | 0.384 |
| Never drinker | 533 (59%) | 54 (70%) |  | 385 (67%) | 34 (59%) |  | 545 (56%) | 113 (62%) |  |
| Former drinker | 171 (18%) | 20 (16%) |  | 110 (14%) | 14 (19%) |  | 249 (27%) | 42 (26%) |  |
| Current drinker | 139 (23%) | 10 (14%) |  | 94 (20%) | 5 (22%) |  | 137 (16%) | 13 (12%) |  |
| **Smoking status** |  |  | 0.144 |  |  | 0.898 |  |  | 0.429 |
| Never smoker | 628 (71%) | 57 (64%) |  | 445 (71%) | 33 (68%) |  | 627 (65%) | 113 (65%) |  |
| Former smoker | 82 (12%) | 7 (7.6%) |  | 54 (9.8%) | 9 (10%) |  | 109 (13%) | 22 (17%) |  |
| Current smoker | 133 (17%) | 20 (28%) |  | 90 (19%) | 11 (22%) |  | 195 (21%) | 33 (18%) |  |
| **History of pelvic infection** |  |  | **0.048** |  |  | **0.022** |  |  | 0.112 |
| Yes | 27 (2.2%) | 8 (5.1%) |  | 24 (4.3%) | 5 (17%) |  | 51 (5.8%) | 17 (11%) |  |
| No | 808 (98%) | 76 (95%) |  | 562 (96%) | 48 (83%) |  | 877 (94%) | 148 (89%) |  |
| **Regular periods** |  |  | **0.007** |  |  | 0.252 |  |  | 0.698 |
| Yes | 793 (96%) | 76 (83%) |  | 556 (94%) | 53 (100%) |  | 875 (94%) | 161 (95%) |  |
| No | 50 (4.5%) | 8 (17%) |  | 33 (6.3%) | 0 (0%) |  | 56 (5.8%) | 7 (4.8%) |  |
| **Depression states** |  |  | 0.219 |  |  | 0.669 |  |  | 0.281 |
| No depression | 661 (77%) | 59 (69%) |  | 439 (73%) | 35 (67%) |  | 640 (69%) | 101 (60%) |  |
| Minimal-to-mild depression | 123 (15%) | 14 (15%) |  | 95 (15%) | 11 (16%) |  | 183 (19%) | 40 (23%) |  |
| Moderate-to-severe depression | 59 (7.5%) | 11 (16%) |  | 55 (11%) | 7 (18%) |  | 108 (12%) | 27 (17%) |  |
| **Sleeplessness** |  |  | 0.502 |  |  | 0.250 |  |  | **0.003** |
| Yes | 156 (19%) | 14 (24%) |  | 101 (21%) | 18 (31%) |  | 238 (27%) | 66 (43%) |  |
| No | 687 (81%) | 70 (76%) |  | 488 (79%) | 35 (69%) |  | 693 (73%) | 102 (57%) |  |
| **History of diabetes** |  |  | **0.007** |  |  | 0.592 |  |  | **0.001** |
| Yes | 9 (0.6%) | 3 (3.9%) |  | 31 (3.5%) | 1 (2.0%) |  | 89 (7.9%) | 29 (18%) |  |
| No | 834 (99%) | 81 (96%) |  | 558 (97%) | 52 (98%) |  | 842 (92%) | 139 (82%) |  |
| **History of hypertension** |  |  | **<0.001** |  |  | 0.980 |  |  | 0.807 |
| Yes | 49 (4.0%) | 11 (17%) |  | 61 (9.9%) | 7 (9.7%) |  | 212 (19%) | 42 (20%) |  |
| No | 794 (96%) | 73 (83%) |  | 528 (90%) | 46 (90%) |  | 719 (81%) | 126 (80%) |  |
| **Serum cotinine (ng/mL)** | 41 ± 102 | 76 ± 138 | 0.552 | 36 ± 90 | 49 ± 103 | 0.526 | 41 ± 94 | 37 ± 92 | 0.607 |
| **Serum vitamin D (nmol/L)** | 70 ± 26 | 72 ± 29 | 0.890 | 67 ± 25 | 73 ± 28 | 0.312 | 56 ± 22 | 56 ± 20 | 0.580 |
| **NHHR** | 1.77 ± 0.70 | 2.02 ± 0.98 | 0.261 | 2.33 ± 1.05 | 2.66 ± 1.04 | 0.068 | 2.92 ± 1.30 | 3.00 ± 1.30 | 0.588 |
| **Physical activity** |  |  | 0.626 |  |  | 0.366 |  |  | 0.880 |
| Insufficient | 221 (19%) | 30 (22%) |  | 171 (23%) | 20 (31%) |  | 303 (30%) | 51 (29%) |  |
| Sufficient | 622 (81%) | 54 (78%) |  | 418 (77%) | 33 (69%) |  | 628 (70%) | 117 (71%) |  |
| **Sedentary behavior** |  |  | 0.568 |  |  | 0.373 |  |  | 0.421 |
| Low sedentary time | 402 (49%) | 40 (53%) |  | 300 (51%) | 22 (42%) |  | 443 (45%) | 79 (48%) |  |
| High sedentary time | 441 (51%) | 44 (47%) |  | 289 (49%) | 31 (58%) |  | 488 (55%) | 89 (52%) |  |
| **Dietary cholesterol (mg)** | 259 ± 175 | 235 ± 156 | 0.482 | 259 ± 162 | 294 ± 164 | 0.289 | 269 ± 164 | 269 ± 190 | 0.340 |
| **Dietary calories (kcal)** | 1,865 ± 673 | 1,787 ± 681 | 0.778 | 1,808 ± 600 | 2,083 ± 662 | **0.016** | 1,881 ± 660 | 1,853 ± 732 | 0.371 |
| **TC (mg/dL)** | 171 ± 32 | 183 ± 32 | **0.008** | 182 ± 34 | 190 ± 36 | 0.281 | 185 ± 36 | 183 ± 36 | 0.508 |
| **HDL-C (mg/dL)** | 64 ± 15 | 65 ± 16 | 0.976 | 58 ± 14 | 54 ± 13 | **0.042** | 50 ± 12 | 49 ± 14 | 0.322 |
| **ALT (U/L)** | 15.9 ± 7.1 | 19.1 ± 8.6 | **0.005** | 19 ± 13 | 19 ± 11 | 0.962 | 23 ± 23 | 22 ± 12 | 0.182 |
| **AST (U/L)** | 20.1 ± 6.4 | 24.0 ± 20.3 | 0.162 | 21 ± 10 | 20 ± 8 | 0.086 | 23 ± 19 | 21 ± 7 | 0.411 |
| **TG (mmol/L)** | 0.97 ± 0.55 | 1.10 ± 0.64 | 0.189 | 1.26 ± 0.75 | 1.54 ± 0.86 | 0.136 | 1.63 ± 2.11 | 1.72 ± 1.16 | 0.239 |
| **UA (umol/L)** | 245 ± 57 | 252 ± 43 | 0.118 | 264 ± 57 | 267 ± 57 | 0.657 | 297 ± 68 | 310 ± 71 | 0.070 |
| **Cr (ng/mL)** | 65 ± 24 | 64 ± 13 | 0.851 | 64 ± 12 | 64 ± 13 | 0.783 | 63 ± 12 | 63 ± 11 | 0.864 |

*P* in bold indicates a significant statistical difference.
